# Assessment of Safety and Functional Outcomes of Arthroscopic Meniscal Repair Surgery Using Surestitch All-Inside Implant

**DOI:** 10.7759/cureus.90520

**Published:** 2025-08-19

**Authors:** Dhammapal S Bhamare, Suhas Kamble, Janapamala V S Kishore, Raj Kamble, Rohan Hajare

**Affiliations:** 1 Orthopaedics, Sainath Hospital, Moshi, IND; 2 Orthopaedics, Visakha Institute of Medical Sciences, Visakhapatnam, IND; 3 Orthopaedics, Dr. D. Y. Patil Medical College, Hospital & Research Centre, Pimpri-Chinchwad, IND

**Keywords:** ikdc score, lysholm score, meniscal tear, sane score, tegner level

## Abstract

Introduction

Meniscal injuries are prevalent in orthopedic practice, affecting a diverse patient population. In recent times, a notable shift has occurred in the treatment approach for meniscal injuries, favoring repair surgery over the traditional method of meniscectomy. Meniscal repair surgery is a crucial intervention to restore knee function and alleviate symptoms, contributing to improved patient quality of life. Therefore, the present study aimed to assess the safety and functional outcomes of the Surestitch all-inside implants in arthroscopic meniscal repair surgery.

Methods

From July 2020 to July 2022, 51 patients underwent arthroscopic meniscal repair surgery at Sai Nath Hospital, Maharashtra, India. Patient-reported outcomes, including the International Knee Documentation Committee (IKDC) subjective score, Single Assessment Numeric Evaluation (SANE) score, Tegner activity level, and Lysholm knee score, were recorded through telephonic follow-ups. Detailed patient characteristics, diagnoses, and surgery details were retrospectively extracted from medical records.

Results

The mean age of the recruited patients was 37.75±11.65 years. About 71% of patients were male (n=36/51). Medial meniscal tear was more prevalent than lateral tear (38 vs. eight patients). The remaining five patients had combined medial and lateral meniscal tears. Patient-reported outcomes showed the mean IKDC, Tegner, and Lysholm scores of 87.49±10.21, 6.03±1.38, and 93.45±9.36, respectively. On comparing the mean SANE scores of affected and unaffected contralateral knees, a significant difference in the scores was observed (86.73±15.94 vs. 97.55±6.81, p<0.05).

Conclusion

Our data suggest favorable and good functional outcomes of the Surestitch all-inside meniscal repair implant in meniscal repair surgery.

## Introduction

The knee is predisposed to injury due to its intricate anatomical structure and functional demands [1]. The knee joint contains a meniscus that plays a vital role in maintaining knee stability, shock absorption, knee filler, load distribution, and lubrication [1]. Meniscus comprises the U-shaped medial and S-shaped lateral meniscus, positioned between the corresponding femoral condyle and tibial plateau [1-3]. These fibrocartilaginous discs are part of the "meniscus-meniscal ligament complex" that forms intricate networks with surrounding ligamentous structures and condyles of the femur and tibia [1-3]. Both menisci are responsible for transmitting >50% of the load during knee movement. For example, at 30° of knee flexion, the contact area between the femoral and tibial surfaces decreases by approximately 4%. The meniscus compensates for abnormalities between the femoral and tibial articular surfaces by providing chondroprotection through enhanced weight distribution [4,5]. In addition, ligaments such as the posterior cruciate ligament (PCL), anterior cruciate ligament (ACL), medial collateral ligament (MCL), and lateral collateral ligament (LCL) collectively support the weight and flexibility of the knee by controlling the knee movements and protecting the knee from overextension [3].

Meniscal or ligament injuries are commonly observed in active individuals, particularly athletes, stemming from sudden traumatic events. Additionally, older individuals may experience such injuries due to gradual wear and tear over time [6]. These injuries can lead to pain and swelling and restrict the range of motion in the knee. The estimated incidence of meniscal tears is 60 per 100,000 people, potentially contributing to early-onset osteoarthritis [3,5]. In the past, meniscal tears were rectified by partial or complete meniscectomy. However, meniscectomy is associated with a 10-year risk of osteoarthritis of 40% in the lateral and 22% in the medial compartments [7]. This emphasizes the importance of considering alternative treatments that may reduce the risk of osteoarthritis development. Therefore, a paradigm shift has been observed in the treatment strategy of meniscal injury from meniscectomy to meniscal repair surgery [8,9].

Meniscal tear surgery is a common procedure in orthopedic practice, performed using inside-out, outside-in, and all-inside techniques [1,10-13]. The choice of surgical technique and implants has evolved, with advancements in arthroscopic procedures and implant technology contributing to better outcomes. Recently, the Surestitch all-inside meniscal repair implant (Healthium Medtech Limited, India), comprising two PEEK (polyether ether ketone) non-absorbable implants pre-tied with USP #2-0 non-absorbable UHMWPE (ultra-high-molecular-weight polyethylene) suture and preloaded into a needle delivery system, was introduced as a suture retention device for the facilitation of endoscopic soft tissue fixation [14].

Based on the above background, we aimed to assess the safety and functional outcomes of arthroscopic meniscal repair surgery performed using the Surestitch all-inside implant.

## Materials and methods

Study design

The present clinical study employed a retrospective, single-center, observational research design. Adherence to ethical standards was ensured by following the regulations outlined in the Declaration of Helsinki, the Medical Device Rules 2017, the International Council for Harmonization of Technical Requirements for Pharmaceuticals for Human Use (ICH), and the International Standard ISO 14155:2020.

Ethical approval

The ethical approval was obtained from the Royal Pune Independent Ethics Committee, Pune, Maharashtra, India. The trial was registered with the Clinical Trial Registry of India (CTRI/2023/04/051982). 

Patients

Patients who underwent arthroscopic meniscal repair surgery at Sainath Hospital in Maharashtra, India, between July 2020 and July 2022 were enrolled in the study. Data of the enrolled patients were collected retrospectively by examining their medical records. Subsequent follow-up assessments of the recruited patients were carried out through telephonic communication. All patients were successfully contacted during the follow-up period, ensuring no loss to follow-up. Verbal consent for study participation was obtained from each patient during the telephone interaction. The inclusion and exclusion criteria for patient selection are summarized in Table 1. 

**Table 1:** Inclusion and exclusion criteria.

Criteria Type	Criterion
Inclusion	Patients aged ≥18 years
Inclusion	Underwent meniscal repair surgery with Surestitch all-inside implant (July 2020-July 2022)
Inclusion	Willing to give informed consent during telephonic follow-up
Exclusion	Traumatic injury to the same knee after meniscus repair
Exclusion	Could not be contacted after three attempts

Objectives

We hypothesized that the Surestitch all-inside implant would be safe and yield favorable functional outcomes due to its design features: two PEEK non-absorbable implants pre-tied with UHMWPE sutures and preloaded in a needle delivery system. Based on this hypothesis, the safety of the Surestitch all-inside meniscal repair implant was reviewed by thoroughly assessing the adverse events that occurred in patients post-surgery. Further, the functional outcomes of the Surestitch implant were assessed by evaluating subjective knee function and knee-specific symptoms using the International Knee Documentation Committee (IKDC) profile, Tegner activity level, Lysholm knee score, and Single Assessment Numeric Evaluation (SANE) score.

Surgical procedures

The condition of the injury in the knee was reviewed by magnetic resonance imaging (MRI), guiding the decision to plan surgery for meniscal tear repair using the Surestitch all-inside meniscal repair implant [14]. During meniscal repair surgery, other associated surgeries, such as ACL and MCL, were also performed by the surgeon. The number of implants employed during meniscal repair surgery varied based on the extent and nature of the knee injury.

Data collection

The baseline data of patients’ characteristics, including sex, age, height, weight, marital status, employment status, smoking, drinking, and level of exercise, along with injury details such as side of injured knee, method of injury, type, size, and zone of rupture or tear, and surgical details including number of implants used, associated surgeries, complications, and reoperations, were collected retrospectively from the patients’ medical records. During telephonic follow-ups, the functional outcomes were assessed by asking questions related to IKDC subjective evaluation, Lysholm knee score, SANE score, and Tegner activity score [14]. Additionally, we compared the SANE scores of affected and unaffected sides of the knee.

Statistical analysis

The statistical analysis of the data was performed using GraphPad software v8.0 (GraphPad Software, Boston, MA, USA). Descriptive statistics, such as mean ± SD or proportions and percentages, were used to report the data. A t-test was employed to compare the mean SANE scores between the affected and unaffected sides of the knee. A significance level of p≤0.05 was considered indicative of statistical significance.

## Results

Participants

A total of 51 patients who underwent meniscal repair surgery between June 2020 and July 2022 at Sainath Hospital, Maharashtra, India, were included in the study. The telephonic follow-up was completed on August 10, 2023. At baseline, the mean age of enrolled patients was 37.75±11.65 years, with 71% male predominance (n=36/51). The remaining 15 patients (29%) were females in the study. The recruited patients had a mean height and weight of 169.76±8.23 cm and 70.16±13.45 kg, respectively.

Of 51 Indian patients, 43 (84.3%) were married and eight (15.7%) were single. Smoking and drinking habits were reported in 11 (21.6%) and nine (17.6%) patients, respectively. Employment status was categorized into full-time workers, part-time workers, and non-workers. Among the patients, 28 (55%) were full-time workers or employees or businessmen, eight (15.7%) were part-time workers or sportspersons, and 15 (29.3%) were non-workers, including students and housewives. Referring to their exercise routine, the majority of the patients were involved in moderate (n=25) and severe (n=16) types of exercise, while 15.7% of patients (n=8/51) reported doing “mild” exercise. Two patients did not have any exercise routine. The demographics of the enrolled patients are given in Table 2.

**Table 2:** Demographic characteristics of the patients.

Characteristics, n (%)	Number of patients (n=51)
Age (years; mean ± SD)	37.75±11.65
Gender	
Male	36 (71)
Female	15 (29)
Height (cm; mean ± SD)	169.76±8.23
Weight (kg; mean ± SD)	70.16±13.45
Ethnicity: Asian	51 (100)
Smoking: yes	11 (21.6)
Drinking: yes	09 (17.6)
Marital status	
Single	08 (15.7)
Married	43 (84.3)
Employment status	
Full working (>8 hours)	28 (55)
Part-time (<5 hours)	08 (15.7)
Not working	15 (29.3)
Occupation	
Business	12 (23.5)
Employee	21 (41.2)
Household	09 (17.6)
Student	04 (7.9)
Sports	05 (9.8)
Exercise routine	
No	02 (3.9)
Mild	08 (15.7)
Moderate	25 (49)
Severe	16 (31.4)

Injury details

About 55% of patients (n=28/51) had an injury in the right knee, while 45% of patients (n=23/51) had a left knee injury, with a diverse range of mechanisms, including daily activities (n=28/51) and sports-related incidents (n=22/51). The mechanism of injury was unknown in only one patient. After the injury, 14 patients (27.5%) reported “severe” pain, 29 patients (56.9%) had “moderate” pain, and eight patients (15.6%) reported “mild” pain in their knees. The mean time since injury to surgery day was 3.18±5.26 months. The details of the injury are given in Table 3.

**Table 3:** Details of the injury.

Characteristics, n (%)	Number of patients (n=51)
Knee injury	
Right	28 (55)
Left	23 (45)
Mechanism of injury	
Daily activities	28 (55)
Sports	22 (43)
Unknown	01 (2)
Pain	
Mild	8 (15.6)
Moderate	29 (56.9)
Severe	14 (27.5)
Time of surgery since injury (months; mean ± SD)	3.18±5.26

Diagnosis and surgery details

The patients enrolled in the study were diagnosed with either medial, lateral, or a combination of both types of meniscal tear. The prevalence of medial meniscal tears was significantly higher than the prevalence of lateral tears (38 vs. eight patients). Additionally, five patients presented with both medial and lateral types of tears. Regarding the orientation of meniscal tears, 14 patients (27.6%) had horizontal tears, nine patients (17.6%) had buckle handle tears, four patients each had radial and longitudinal tears, and 10 patients each had vertical and complex tears. The mean length of the tear was 2.33±0.86 cm. About 38 patients exhibited a red-red or red-white zone of rupture, whereas the remaining 25.4% (n=13/51) had both red-red and red-white zones of rupture.

Surgeries for meniscal repair were performed using the Surestitch all-inside implant. Depending upon the surgical details of the patients, two to five implants were used in the surgery. The mean number of days hospitalized was 3.5±1 days. Associated surgeries such as ACL, MCL, and others were also performed in 31, three, and three patients, respectively. The details of the diagnosis and surgery are given in Table 4.

**Table 4:** Details of the diagnosis and surgery. ACL, anterior cruciate ligament; MCL, medial collateral ligament

Characteristics, n (%)	Number of patients (n=51)
Meniscal tear	
Medial	38 (75)
Lateral	08 (15.7)
Both	05 (9.8)
Type of meniscal tear	
Horizontal	14 (27.6)
Vertical	10 (19.6)
Radial	04 (7.8)
Buckle handle	09 (17.6)
Longitudinal	04 (7.8)
Complex	10 (19.6)
Length of tear (cm, mean ± SD)	2.33±0.86
Zone of rupture	
Red-red	19 (37.3)
Red-white	19 (37.3)
Both	13 (25.4)
No. of days hospitalized (days, mean ± SD)	3.5±1
Associated surgeries	
ACL	31 (60.8)
ACL + MCL	03 (5.9)
Others	03 (5.9)

Patient-reported outcomes

The patients were followed up for a mean duration of 24.72±6.19 months. No adverse events or complications were reported by any of the patients during the study. The patient-reported outcome data are summarized in Table 5.

**Table 5:** Patient-reported outcomes after meniscal repair. IKDC, International Knee Documentation Committee; SANE, Single Assessment Numeric Evaluation

Scores, n (%)	Number of patients (n=51)
IKDC score (mean±SD)	87.49±10.21
>90	22 (43)
80-89	15 (29.5)
<80	14 (27.5)
Lysholm score (mean±SD)	93.45±9.36
95-100	27 (53)
84-94	22 (43)
<84	02 (4)
SANE score (mean±SD)	
Affected knee	86.73±15.94
Non-affected knee	97.55±6.81
Tegner activity level (mean±SD)	6.03±1.38
3	3 (5.9)
4	3 (5.9)
5	8 (15.7)
6	25 (49)
7	6 (11.8)
8	3 (5.9)
9	2 (3.9)
10	1 (1.9)

Subjective Knee Function and Knee-Specific Symptoms

During the telephonic follow-up, the patients reported a mean IKDC score of 87.49±10.21. Among these recruited patients, 22 patients (43%) achieved IKDC scores of 90 or above, indicating favorable knee function, 15 patients (29.5%) in the range of 80-89, and the remaining 14 patients (27.5%) had scores below 80.

**Figure 1:**
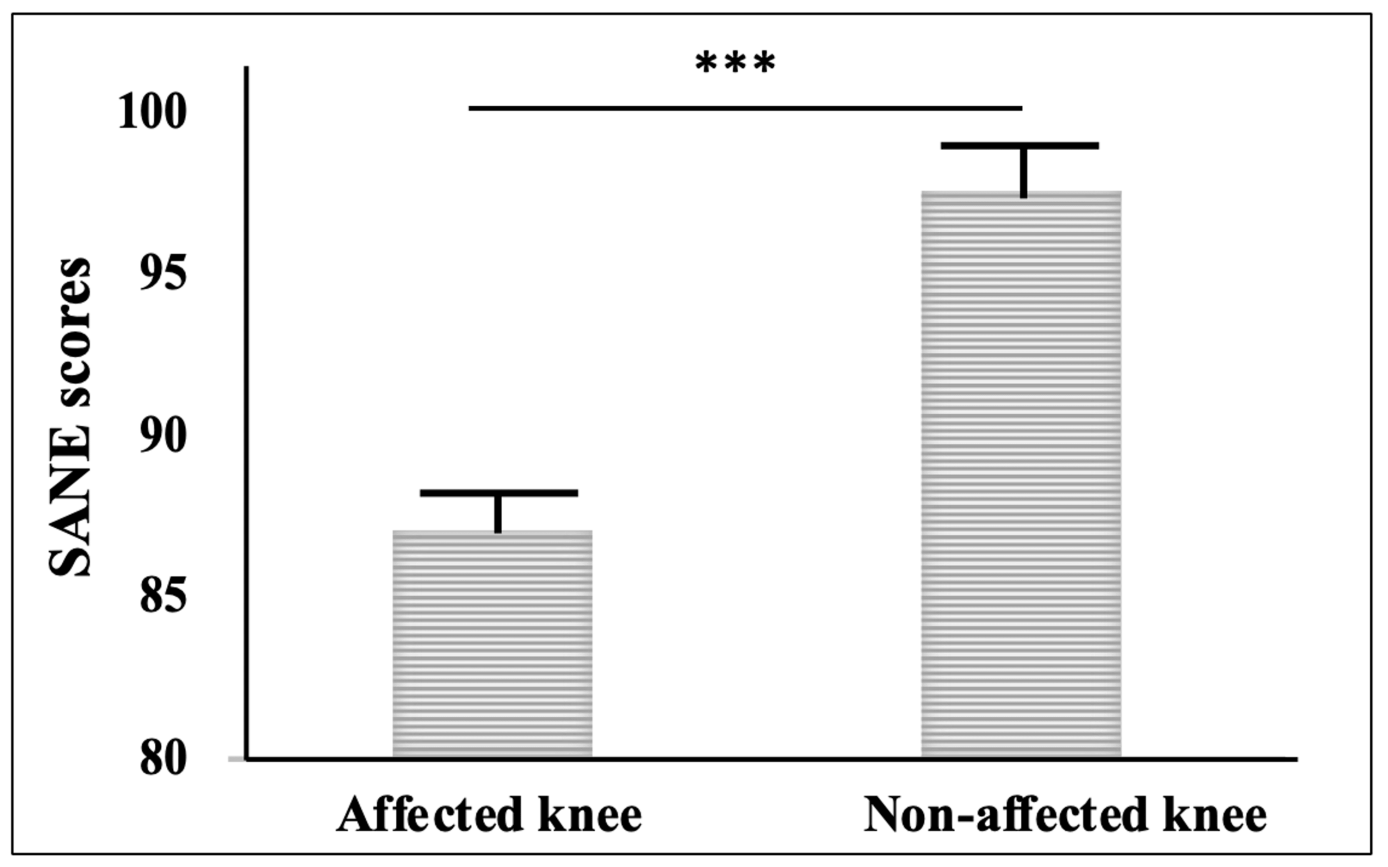
SANE score: bar chart representing and comparing the SANE score in affected and non-affected knees. *** p<0.0001 SANE, Single Assessment Numeric Evaluation

The Lysholm score of the patients was found to be 93.45±9.36. A majority of patients (53%, n=27/51) achieved excellent scores in the range of 95 to 100, followed by 43.1% (n=22/51) with good scores in the range of 84 to 94. Only two patients had Lysholm scores below 84. Furthermore, the patients were asked to score their affected and non-affected knees. The mean SANE score of the affected knee was 86.73±15.94, which was significantly lower than the SANE score of the other knee (unaffected knee: 97.55±6.81, p<0.0001). The SANE scores are shown in Figure 1.

Tegner Knee Activity

The activity level of the patients after surgery was assessed using the Tegner scale. The mean Tegner score of the enrolled patients was 5.98±1.42, with scores ranging from 3 to 10. About 25 patients (49%) had a Tegner activity level of 6. 

## Discussion

The present study focused on the safety and functional outcomes of meniscal repair surgery using the Surestitch all-inside meniscal repair implants in a cohort of 51 patients. The main finding of the study is that the Surestitch all-inside implant provides favorable functional outcomes of meniscal repair surgery. Notably, all-inside repair implants are faster and minimally invasive compared to inside-out and outside-in implants [14].

Patient heterogeneity is one of the prime aspects to consider in a study. In this study cohort, the age of the recruited patients ranged between 18 and 67, covering all the patients from different age groups. Several studies have been conducted in the past considering age as an important factor to seek the outcomes of meniscal repair surgery; however, it is not found as a contraindication to meniscal repair [15-18]. For instance, Engler et al. (2022) recruited patients aged 40 years and older who underwent meniscal repair or meniscectomy and showed good results with reference to patient-reported outcome measures [15]. Another study conducted by Husen et al. (2022) reported benefits and favorable outcomes of meniscal repair in patients older than 60 years [16]. The range of ages in this study aligns with the findings of Panchal et al. (2023) [14] and Ventura et al. (2023) [17]. The demographic characteristics of the enrolled patients align with existing literature on meniscal injuries and repair surgeries.

Evidence in the literature showed that medial tears are more common than lateral injuries [16,17,19]. We also found that about 85% of recruited patients had medial meniscal tears in our study. Our data are consistent with the findings of Buchbinder et al. (2016) [19], Husen et al. (2022) [16], and Ventura et al. (2023) [17], where the majority of patients had medial meniscus tears. The predominance of medial meniscal tears is consistent with previous reports, highlighting the importance of understanding tear patterns for effective surgical planning [20].

Considering patient-reported outcome measures, we observed an IKDC score of 87.49 and a SANE score of 86.73 in our study. Similar to our data, Laurendon et al. (2017) determined prognostic factors for failure of an all-inside meniscal repair and reported a mean IKDC score of 88.19 after a follow-up of 31 months [21]. Contrary to our study, Panchal et al. (2023) reported a lower IKDC score of 81.72 and a higher SANE score of 94.02 using the Surestitch all-inside implant. The variation in scores may be attributed to the disparity in follow-up durations (25 months versus 16 months), indicating that a longer post-surgery timeframe is associated with differential scores. Furthermore, in our study, the postoperative mean Lysholm score was 93.45, which aligns with the findings of Abdallah et al. (2020) following meniscal repair using different arthroscopic techniques [22].

Finally, the observed discrepancy in SANE scores between affected and unaffected knees is a common finding post-meniscal repair. Patients may perceive a difference in function between the two knees, even if the surgical outcome is deemed successful. This emphasizes the importance of comprehensive preoperative counseling to manage patient expectations and enhance postoperative satisfaction [23].

Limitations

The study acknowledges several limitations. The study is based on a retrospective design with a small sample size. Post-operative rehabilitation has a potential role in patient recovery and functional outcomes. However, the rehabilitation factor could not be considered due to the retrospective study design. Furthermore, the success of the Surestitch implant could not be compared with a control group, limiting the ability to conclude the superior efficacy of this implant.

## Conclusions

In conclusion, the present study indicates that arthroscopic meniscal repair with the Surestitch all-inside meniscal repair implant leads to satisfactory and favorable functional outcomes in patients. Patient-reported outcomes provide sufficient evidence on the effectiveness of the implant in promoting the well-being of the patients. This research would help clinicians and patients to make informed decisions about the Surestitch all-inside meniscal repair implant as an appropriate treatment option for the surgery.
